# Changes in expression of Wnt signaling pathway inhibitors dickkopf-1 and sclerostin before and after total joint arthroplasty

**DOI:** 10.1097/MD.0000000000008082

**Published:** 2017-09-29

**Authors:** Ke Lu, Yi-Xuan Li, Tian-Shu Shi, Fei Yu, Si-Cong Min, Liang Qiao, Lan Li, Hua-Jian Teng, Qing Jiang

**Affiliations:** a Department of Sports Medicine and Adult Reconstructive Surgery, Drum Tower Hospital, School of Medicine; b Joint Research Center for Bone and Joint Disease, Model Animal Research Center (MARC), Nanjing University, Nanjing, Jiangsu, China.

**Keywords:** osteoarthritis, serum dickkopf-1, serum sclerostin, total joint arthroplasty

## Abstract

The aim is to study how serum concentration of Dickkopf-1 (DKK1) and Sclerostin (SOST) varies in patients before and after undergoing total joint arthroplasty (TJA). A total of 104 patients undergoing TJA were included in this study. Serum DKK1 and SOST were measured at 1 day before and 1, 3, and 5 days after surgery. DKK1 levels were highest at 5 days’ postoperation, increasing to 25.17% above preoperation levels (*P* < .01), while SOST levels were lowest at 3 days’ postoperation, falling to 18.71% below preoperation levels (*P* < .05). Serum levels of DKK1 and SOST showed opposite trends in the days following TJA. Our research describes for the first time the perioperative changes observed in serum DKK1 and SOST levels of osteoarthritis (OA) patients undergoing TJA. Increased DKK1 and decreased SOST levels may help maintain the equilibrium of the WNT pathway in OA patient's postsurgery.

## 1 Introduction

Osteoarthritis (OA) is a common disease of the joints with symptoms often including pain, stiffness, swelling, reduced activity levels, and deformities. The vast majority of OA cases are caused by a progressive degeneration of joint cartilage, with 4 key pathological manifestations: joint space narrowing, the formation of osteophytes, subchondral sclerosis, and synovitis.^[1]^ Although the precise nature of the causes and pathogenesis of OA has not yet been fully explained, it is currently believed that the disease is linked with age, weight, and other factors.^[2]^ A number of biochemical factors play an important role in the incidence and development of OA.

The regulatory effect of the Wnt/β-catenin signaling pathway on osteoblast and chondroblast metabolism has recently become a hot topic of research.^[3]^ We are now aware that Dickkopf-1 (DKK1) and sclerostin (SOST) are both upstream inhibitors of the Wnt/β-catenin pathway, and that both can negatively influence bone mass.^[4,5]^ Diarra et al^[6]^ inhibited DKK1 expression in a mouse model of rheumatoid arthritis. Uderhardt^[7]^ also inhibited DKK1 expression in mice, inducing sacroiliac joint fusion. SOST is a protein produced primarily by osteocytes and hypertrophic chondrocytes, and SOST mutation can lead to sclerosteosis and van Buchem disease.^[8]^

In clinical settings, a large body of research has measured OA patients’ relevant serological indicators to assess the severity of illness and to serve as a reference point for joint replacement surgery. There have already been reports on serum concentration of DKK1 and SOST in OA patients, and there is literature indicating that serum concentration of DKK1 and SOST are negatively correlated with assessed OA severity score.^[9,10]^ Both DKK1 and SOST levels are lower in OA patients than in healthy subjects, and levels continue to decrease as OA severity increases.^[9–11]^ Still, there has been no literature discussing changes in DKK1 and SOST levels of total joint arthroplasty (TJA) patients during the perioperative period. Our report is the first to compare levels of Wnt/β-catenin signaling pathway inhibitors in a Chinese cohort before surgery and at 1, 3, and 5 days after surgery. This research will help further our understanding of the role the Wnt/β-catenin pathway plays in OA pathology, and will serve as both a reference and a guideline for future TJA surgeries.

## 2 Methods

### 2.1 Subjects

Subjects were patients undergoing surgery at the Department of Sports Medicine & Adult Reconstructive Surgery at Nanjing Drum Tower Hospital between February 2014 and June 2015. A total of 104 patients diagnosed with OA, including 23 males and 81 females were enrolled in the study. Mean age was 68.7981 ± 9.5935 years. The percentage of subjects undergoing TKA was 49.0%, with the rest undergoing THA. Exclusion criteria were as follows: older than 80 years; diagnosed with hypertension, diabetes mellitus, autoimmune diseases, parathyroid or renal diseases; use of glucocorticoid hormone within the last 3 months; history of infection; and previous joint replacement. The study was approved by the ethics committee of Drum Tower Hospital (2016-200-01), and all patients provided written informed consent.

### 2.2 Lab tests

Blood samples were taken at 1, 3, and 5 days after surgery. All samples were obtained between 07:00 and 09:00 using disposable ethylenediaminetetraacetic acid (EDTA) vacuum blood collection tubes and tested after over 8 hours. Samples were then centrifuged at 1800 *g* for 10 minutes to separate the serum and stored at -80°C until analyzed. Serum DKK1, SOST, and hyperparathyroidism were measured using MILLIPLEX MAP Human High Sensitivity Cytokine/Chemokine Panel Kits (EMD Millipore, Billerica, MA, Cat. No. HBNMAG-51K) according to manufacturer's instructions. No missing data were reported.

### 2.3 Statistical analysis

Statistical analysis was performed using SPSS v22.0 (SPSS Inc., Chicago, IL) and graphs were done with GraphPad Prism v7.0 (GraphPad Software Inc., La Jolla, CA). Data were analyzed with repeated-measure analysis of variance (ANOVA). A *P* value lower than .05 was considered statistically significant.

## 3 Results

This study included a total of 104 patients undergoing TJA, with a male to female ratio of 23/81 and an average age of 68.79 years. Fifty-one subjects underwent TKA, while the remaining 53 underwent THA. According to the Kellgren and Lawrence (KL) classification, 25 patients were KL grade 2 OA, 45 patients were KL grade 3, and 34 patients were KL grade 4. Serum concentration of DKK1 and SOST was measured at 1 day before and 1, 3, and 5 days after surgery.

To explore the nature of the trends in DKK1 and SOST levels of OA patients after surgery, we assessed serum concentration of these biomarkers at 1, 3, and 5 days post-op (Table 1).

**Table 1:**
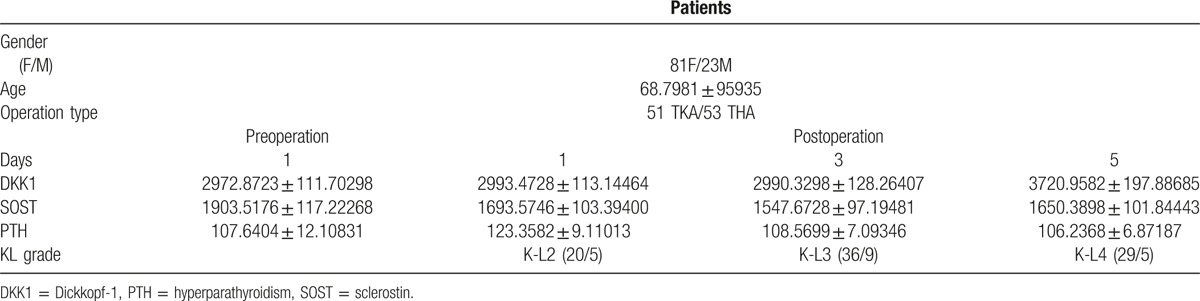
Clinical characteristics of study patients and serum biomarker concentrations.

**Figure 1:**
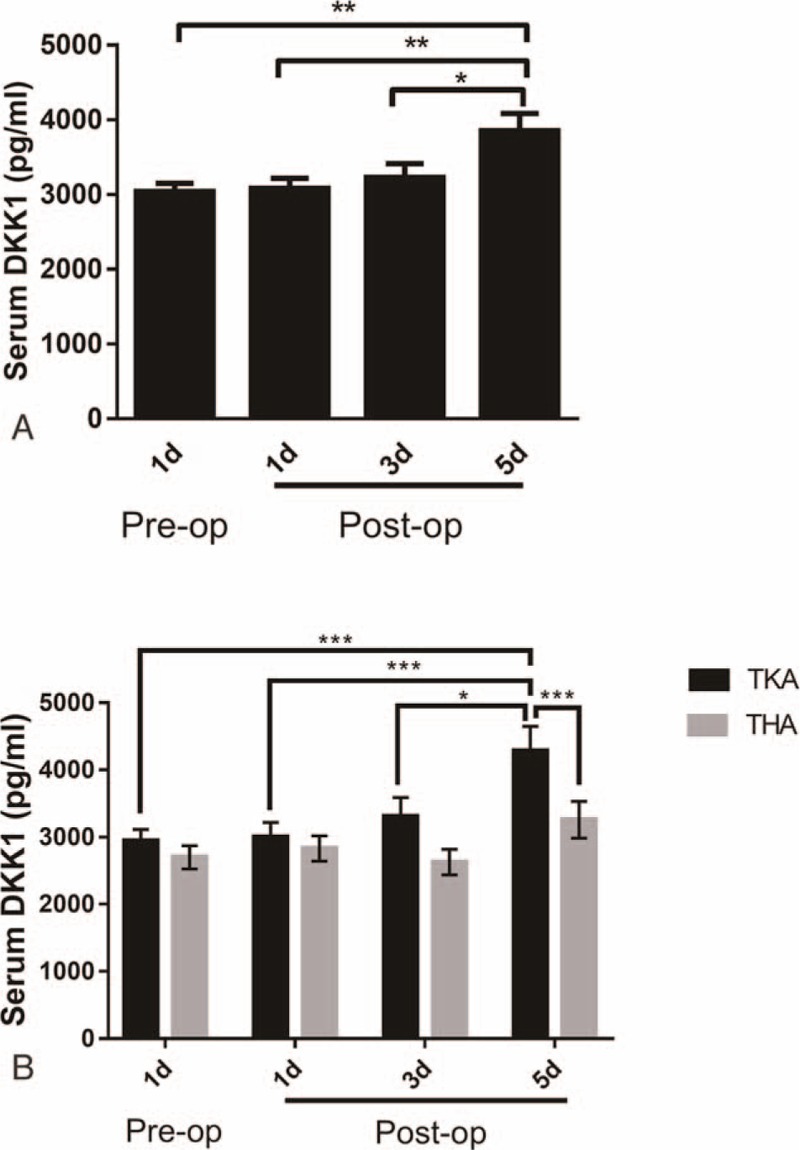
(A) Serum DKK1 levels of OA patients pre- and postoperation. (B) Serum DKK1 levels of TKA or THA patients pre- and postoperation.

The average DKK1 level before surgery was 2972.8723 ± 111.70298 pg/mL. After TJA, DKK1 levels increased gradually as time progressed. At 1 day post-op, the average DKK1 level was 2993.4728 ± 113.14464 pg/ml, with an increase of 0.7% above the pre-op baseline, and remained approximately constant at 3 days post-op. At 5 days post-op, OA patients’ DKK1 levels reached a maximum of 3720.9582 ± 197.88685 pg/mL, showing a noticeable, statistically significant (*P* < .01) increase of 25.17% above baseline (Fig. 1A). We also analyzed serum DKK1 in TKA and THA patient, respectively, and found that serum DKK1 in TKA at 5 days post-op was significantly higher than that in THA patient (Fig. 1B).

**Figure 2:**
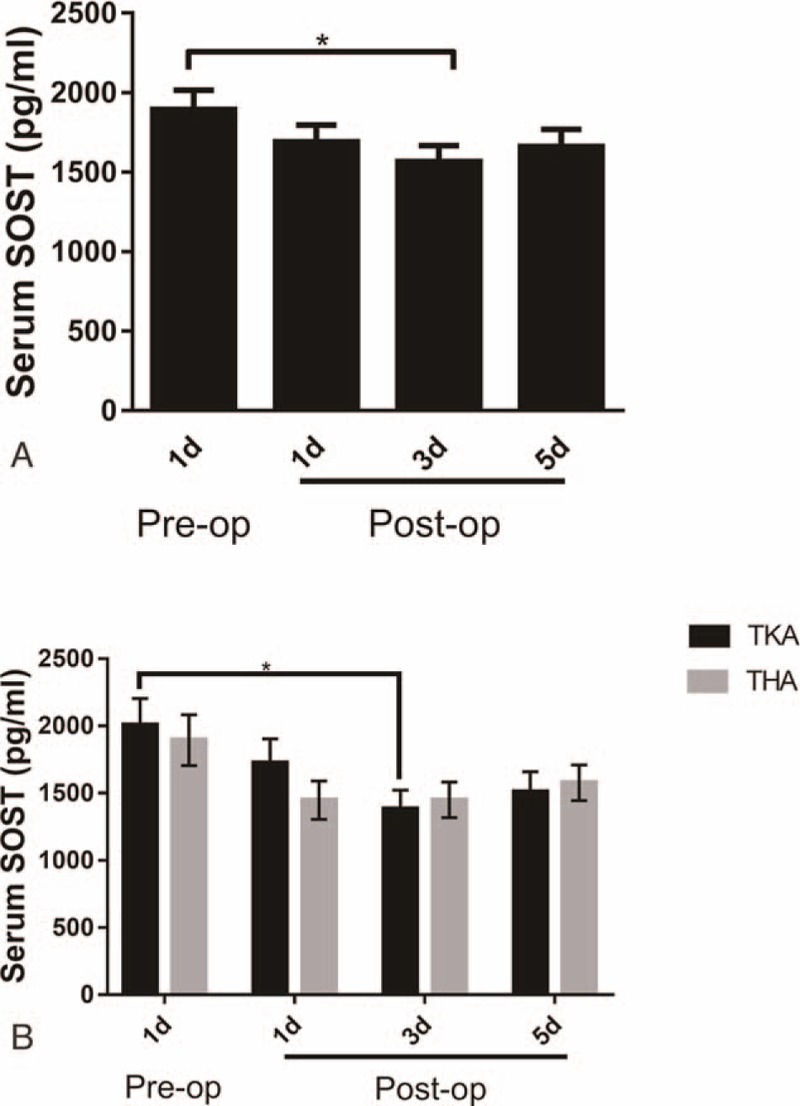
(A) Serum SOST levels of OA patients pre- and postoperation. (B) Serum SOST levels of TKA or THA patients pre- and postoperation.

**Figure 3:**
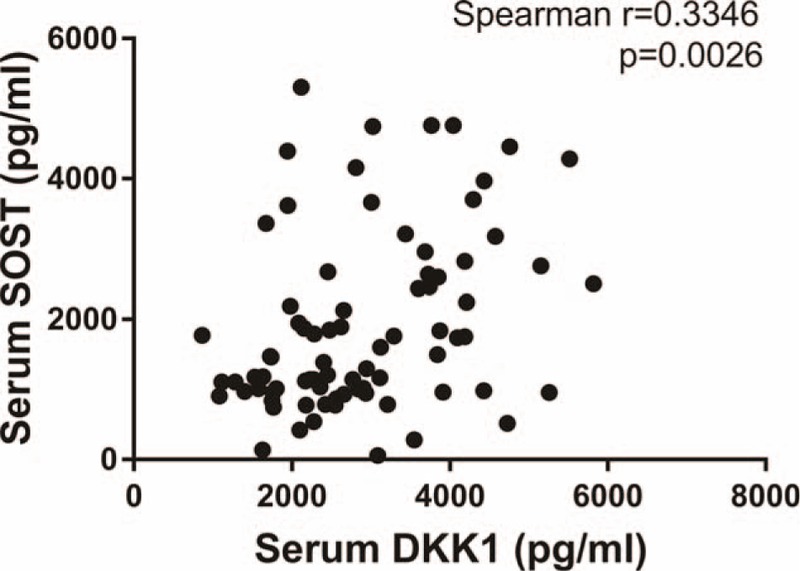
Correlation between serum DKK1 levels and SOST levels of patients before TJA.

**Figure 4:**
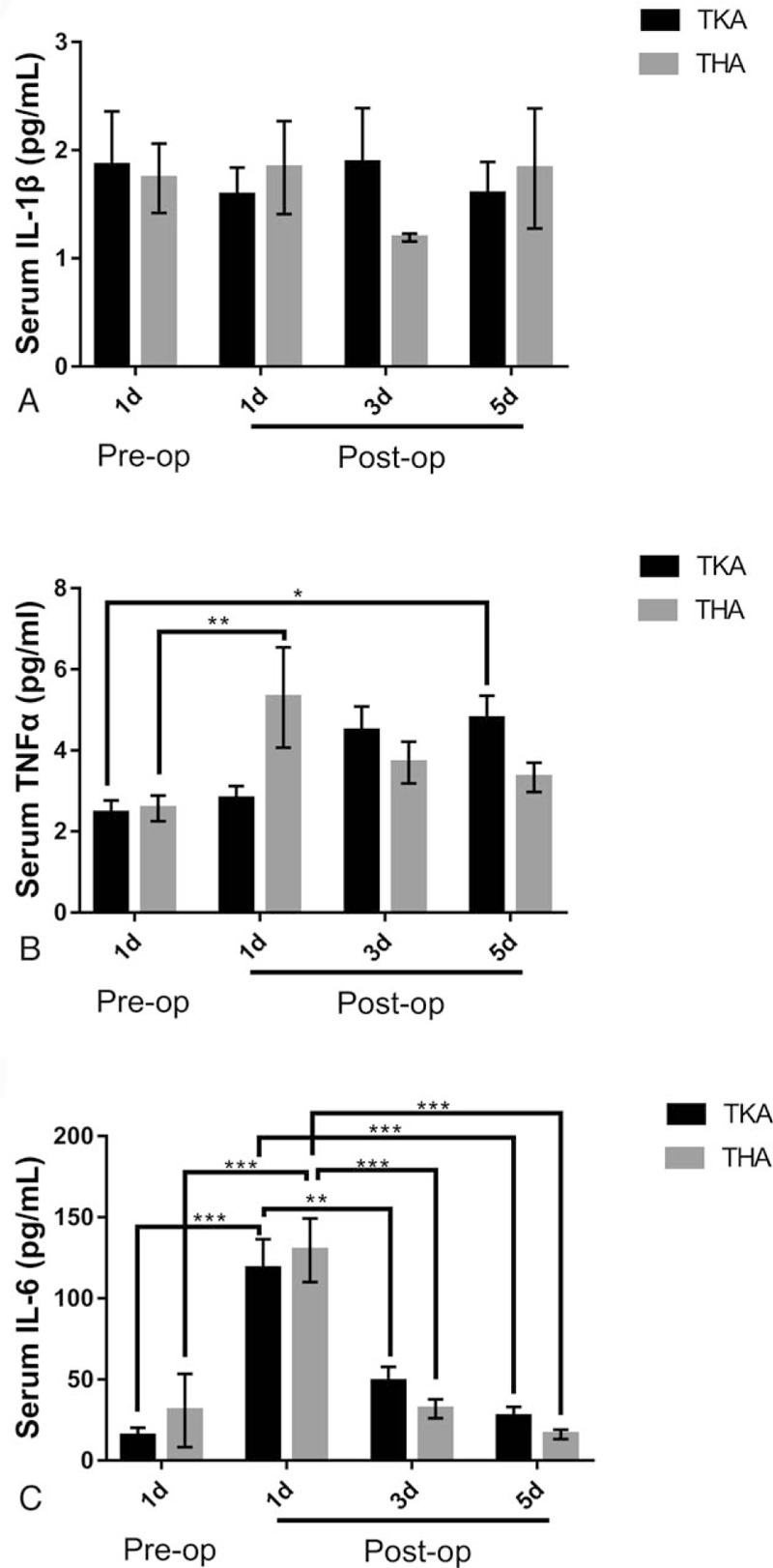
Serum IL-1β (A), TNFα (B), and IL-6 (C) levels of OA patients pre- and postoperation.

In contrast with the trend seen in DKK1 levels, the average pre-op SOST level was 1903.5176 ± 117.22268 pg/mL, dropping, though not statistically (*P* > .05), to 1693.5746 ± 103.39400 pg/mL at 1 day post-op. At 3 days post-op levels continued to drop, falling by 18.71% to an average of 1547.6728 ± 97.19481 pg/mL, a statistically significant (*P* < .05) difference compared with the pre-op baseline. At 5 days post-op, levels showed a slight upturn, rising to 1650.3898 ± 101.84443 pg/mL but still below the pre-op baseline (Fig. 2A). And in TKA patients, 3 days’ post-op levels were also lower than those of the pre-op group but serum SOST showed no difference in THA patients (Fig. 2B). Interestingly, serum DKK1 levels were slightly positively correlated with serum SOST levels before TJA (Fig. 3). Meanwhile, some inflammatory factors such as interleukin (IL)-1β, tumor necrosis factor alpha (TNFα), and IL-6 were detected as well. We found that IL-1β showed no significant difference pre- and postoperation, while TNFα and IL-6 had a bell-shaped change that requires more explanations (Fig. 4).

## 4 Discussion

TJA is a surgical operation used to treat moderate and severe OA where the severely damaged joint is replaced with an artificial joint prosthesis to relieve pain and restore joint function.^[12]^ In the United States, approximately 1 million new patients undergo TJA every year.^[13]^ Corresponding data regarding the situation lack in China, but TJA is a routine procedure frequently carried out in Chinese hospitals. Focusing on the postoperative period is therefore an important part of learning more about OA.

Biochemical markers are key measurement indicators of physiological changes in the body, for both the progression of disease and the course of pharmaceutical or surgical treatment.^[14]^ Plenty of current research has explored changes in relevant markers for OA and joint arthroplasty in order to achieve preventative, treatment, and diagnostic goals. There has also been a large deal of research focusing on changes in TJA-related factors. For example, 1 paper studied the changes in OPN, RANKL, OCN, and ALP expression in the year following TJA surgery, finding that even at 8 months after surgery, OPN, OCN, and ALP levels were still higher than their pre-op baseline.^[15]^ Lawrence et al^[16]^ measured changes in serum ICTP (cross-linked carboxyterminal telopeptide of type I collagen) in patients who had undergone THA and found that ICTP was an indicator of aseptic prosthesis loosening. Other research has focused on the relationship between OA severity and certain biochemical factors. Wisniewski et al^[17]^ determined that tumor necrosis factor-stimulated gene-6 is an independent biomarker for both the progression of OA and the need for TJA.

The Wnt signaling pathway has an important effect on osteoblast differentiation.^[3]^ One of the better researched Wnt pathway inhibitors is DKK1, which can inhibit osteoblast differentiation through its effect on the Wnt pathway.^[6]^ Clinical studies have found that serum DKK1 concentration in patients with multiple myeloma is higher than that in normal control groups,^[18]^ while in patients suffering from hip arthritis, decreased expression of DKK1 was found to slow the rate of joint degeneration.^[19]^ Oh et al^[20]^ used destabilization of the medial meniscus (DMM) to model OA in mice and found that *Col2a1-Dkk1*-transgenic mice exhibited milder OA phenotypes than the control group. Similarly, SOST is also an inhibitor of the Wnt pathway.^[21]^ SOST knockout mice exhibit osteosclerosis and noticeable increased bone mass.^[22]^ In humans, SOST levels are age-dependent, tending to increase with age, and postmenopausal women have noticeable elevated SOST levels compared with women before menopause.^[23,24]^ As both DKK1 and SOST are Wnt pathway inhibitors and secretory proteins, serum concentration of these proteins is often measured to assess their degree of expression in the bone microenvironment. Research in this area has demonstrated that, compared with DKK1, SOST is more selective in the inhibition of the Wnt pathway.^[25]^

Our research has shown that DKK1 and SOST, as important Wnt signaling pathway inhibitors, exhibit opposite trends in OA patients before and after TJA. DKK1 levels rise gradually during postoperative recovery, reaching a maximum at 5 days post-op. On the contrary, SOST levels were found to decrease, reaching a minimum at 3 days post-op. This may cause the constitutive activation of the Wnt signaling pathway. There have already been reports showing that as OA severity increases, SOST and DKK1 levels in blood serum and synovial fluid tend to decrease.^[9,10]^ However, no reports have focused on short-term changes in SOST and DKK1 levels of TJA patients during the perioperative period. This study described changes in SOST and DKK1 levels of patients before and at 1, 3, and 5 days after surgery, providing guidance in some degree for perioperative recovery of TJA patients. As for why the 2 biomarkers exhibited opposite trends, or why DKK1 levels increased while SOST levels decreased, additional research and hypotheses are needed to fully answer these questions, which will also provide us with a deeper understanding of the role the Wnt pathway plays in chondroblast and osteoblast metabolism.

## Acknowledgment

We thank the patients and their families who donated their blood samples for this study.
